# Ovarian pregnancy: clinical characteristics, diagnostic challenges, and sonographic features—a multicenter case series with narrative literature overview

**DOI:** 10.1007/s00404-026-08460-w

**Published:** 2026-05-13

**Authors:** Adi Dayan-Schwartz, Ariel Zilberlicht, Nadav Cohen, Fahoum Leen, Suzan Abd Elgani, Liron Kogan, Ari Reiss, Ronit Beck-Fruchter, Etty Daniel-Spiegel

**Affiliations:** 1 Rappaport Faculty of Medicine, Technion-Israel Institute of Technology, Haifa, Israel; 2 Department of Obstetrics and Gynecology, Emek Medical Center, Afula, Israel; 3 Division Reproductive Endocrinology, Lady Davis Carmel Medical Center, Haifa, Israel; 4 Department of Obstetrics and Gynecology, Carmel Lady Davis Medical Center, Haifa, Israel; 5 Hadassah Medical Center, Faculty of Medicine, Hebrew University of Jerusalem, Jerusalem, Israel; 6 Department of Obstetrics and Gynecology, Clalit Health Services, North District, Israel

**Keywords:** Early pregnancy, Ectopic pregnancy, Ovarian pregnancy, Transvaginal ultrasound, Endometrium

## Abstract

**Purpose:**

To describe the demographic, clinical, laboratory, imaging, and surgical features of 15 histologically confirmed ovarian pregnancies (OP) and to contextualize these findings within a narrative literature overview in order to improve clinical recognition and facilitate earlier diagnosis.

**Methods:**

A retrospective multicenter case series was conducted across two university-affiliated hospitals between 2012 and 2024. Women with histologically confirmed OP were included. Demographic data, risk factors, presenting symptoms, β-hCG dynamics, ultrasound findings, operative details, and postoperative outcomes were collected and analyzed.

**Results:**

Fifteen patients were identified. The mean age was 34.6 ± 4.2 years; 46.7% used an intrauterine device, 33.3% had prior cesarean delivery, and 13.3% conceived through assisted reproduction. Abdominal pain was the predominant symptom (86.7%), whereas vaginal bleeding occurred in 26.7%. The mean preoperative β-hCG level was 6,436 ± 5,570 mIU/mL and serial measurements showed inappropriate rises. OP was suspected preoperatively in 53.3% of cases; identification appeared higher in cases with formal ultrasound (85.7%), although this observation is limited by differences in imaging setting and documentation.. Observed sonographic features included a hyperechoic peripheral ring and a Doppler pattern demonstrating a single dominant feeding vessel; A trilaminar endometrial pattern was not observed in evaluable cases. Rupture occurred in 73.3% of patients with a median blood loss of 300 mL (IQR 10–2000 mL). All patients were treated surgically with ovarian preservation, and postoperative day-1 β-hCG declined by 59 ± 12%.

**Conclusion:**

OP commonly presents with abdominal pain and minimal bleeding and carries a high rupture risk. The described sonographic patterns may represent hypothesis-generating observations that could support clinical suspicion and warranting further study.

**Supplementary Information:**

The online version contains supplementary material available at 10.1007/s00404-026-08460-w.

## What does this study adds to the clinical work?

**Table:** 

This study describes sonographic features observed in ovarian pregnancy, including a single dominant Doppler vessel and absence of a trilaminar endometrium, which may assist clinical suspicion and warrant further study. Higher identification rates were observed with formal ultrasound compared to emergency assessment.	

## Introduction

Ovarian pregnancy (OP) is a rare ectopic pregnancy (1–3% of cases [1–3]) that often mimics tubal ectopic pregnancy (TEP), leading to delayed diagnosis and morbidity. Misdiagnosis or delayed recognition may lead to rupture, hemoperitoneum, and substantial morbidity.

Risk factors for OP are similar to those for other ectopic pregnancies and include intrauterine device (IUD) use and assisted reproductive technologies (ART) [3–5].

Advances in transvaginal ultrasound have improved preoperative detection, yet diagnostic accuracy remains variable. While some studies report correct preoperative diagnosis in up to 75% of cases, misclassification as TEP remains common. Sonographic differentiation from hemorrhagic corpus luteum (CL) is particularly challenging, and standardized diagnostic markers are lacking [6, 7].

The existing literature is dominated by case reports and small series, with limited systematic evaluation of sonographic and clinical features. Specifically, the role of endometrial patterns, Doppler flow characteristics, and β-hCG dynamics in OP diagnosis has not been fully clarified.

The objective of this study was to describe the demographic, clinical, laboratory, imaging, and surgical characteristics of 15 histologically confirmed OPs, and to contextualize our findings within a narrative literature overview.

## Methods

We conducted a retrospective multicenter case series of patients diagnosed with OP between 2012 and 2024 at two university-affiliated teaching hospitals. Institutional Review Board (IRB) approval was obtained at each site (Emek Medical Center #EMC-0069-24, April 2024; Carmel Medical Center #CMC-0173-24, December 2024). Patient consent was not required as this retrospective study included only de-identified data.

Cases were identified using ICD-9 coding for ectopic pregnancy and confirmed through surgical and pathology reports. Inclusion required histologic confirmation of trophoblastic tissue within ovarian tissue. Patients with suspected OP but lacking histologic confirmation were excluded.

Demographic, obstetric, and medical histories were extracted from electronic medical records, along with sonographic, laboratory, and intraoperative findings. Imaging data were reviewed where available. Ultrasound findings were categorized by presence of a yolk sac, endometrial thickness and pattern, ovarian morphology, pelvic fluid, and Doppler features. β-hCG levels were recorded at presentation, during follow-up, and after surgery. Surgical outcomes included rupture status, hemoperitoneum, blood loss, and ovarian preservation.

Sonographic definitions:

- A “single dominant vessel”—Focal Doppler signal of a single prominent feeding vessel supplying only part of the lesion.
- A “trilaminar endometrium”—Three-layer pattern consisting of a central echogenic line with surrounding hypoechoic and outer echogenic layers.
- Formal ultrasound examinations were performed in dedicated obstetrics and gynecology units with stored images, whereas emergency assessments were based on bedside ultrasound reports without standardized image storage.

Data availability varied across variables due to differences in imaging documentation and clinical setting.

### Statistical analysis

Continuous variables are reported as mean ± standard deviation (SD) or as median with range where appropriate, while categorical variables are presented as frequency and percentage. Given the descriptive nature of this case series, statistical analysis was confined to summary statistics, and no formal hypothesis testing was performed.

## Results

Fifteen patients with histologically confirmed OP were included. The mean age was 34.6 ± 4.2 years, and the mean BMI was 24.7 ± 5.9 kg/m^2^. All patients were nonsmokers, and none had a history of pelvic inflammatory disease. The mean gravidity was 4.1 ± 1.8, with a mean parity of 2.4 ± 1.4. A prior cesarean delivery was documented in 5 patients (33.3%), and 1 patient (6.7%) had a history of TEP.

Two pregnancies (13.3%) followed ART; one was the result of transfer of a single day-5 frozen blastocyst under hormonal support, while for the second, no ART details were available. An IUD was present in 7 patients (46.7%): 4 copper, 1 Mirena, 1 Kyleena and 1with unknown type,with a mean duration of use of 2.8 ± 2.0 years (range 0.25–5). Detailed demographic and medical history are summarized in Table 1.

**Table 1:** Demographic and Medical History

Variable	(*N* = 15)
Age (years)	34.6 ± 4.2
Ethnicity	Jewish 9 (60%), Arab 6 (40%)
BMI (kg/m^2^)	24.7 ± 5.9
Smoking	0
PID history	0
Gravida	4.1 ± 1.8
Parity	2.4 ± 1.4
Previous cesarean section	5 (33.3%)
Previous ectopic pregnancy	1 (6.7%)
ART conception	2 (13.3%)
Intrauterine device (IUD) in situ	7 (46.7%): Copper 4, Mirena 1, Kyleena 1, Unknown type 1
Duration of IUD use (years)	2.8 ± 2.0 (range 0.25—5)

*BMI* body mass index, *PID* pelvic inflammatory disease, *ART* assisted reproductive technology, *IUD* intrauterine device

Results are presented as mean ± SD or n (%) when appropriate

The mean gestational age at surgery was 5.9 ± 1.3 weeks. The mean preoperative serum β-hCG was 6436.1 ± 5570.4 mIU/ml. Abdominal pain was the predominant presenting symptom (13, 86.7%), while vaginal bleeding was less frequent (n = 4, 26.7%). Mean preoperative hemoglobin was 11.9 ± 1.4 g/dL.

Imaging availability varied across cases. Formal ultrasound (performed in a dedicated Obstetrics and Gynecology unit with stored images) was available in 7/15 cases, and Doppler data in 6/15 cases. Endometrial characteristics were documented in 13/15 cases, including reports based on textual descriptions without available images. All cases had ultrasound documentation available in the form of written reports. Transvaginal ultrasound demonstrated endometrial thickness ≥ 10 mm in 9 of 13 cases with endometrial documentation (69.2%), but none exhibited a trilaminar pattern (13/13). In several cases, IUD presence or intrauterine fluid obscured the endometrial pattern. A yolk sac was visible in 9 cases (60%); in 4 of these, the initial impression was TEP. All patients with available sonographic imaging (7/7) demonstrated a hyperechoic circular ring at the lateral aspect of the ovary, and in 5 cases a CL was noted in addition to this finding.

Detailed availability and reasons for missing or non-evaluable data are summarized in Supplementary Table 1.

**Fig. 1:**
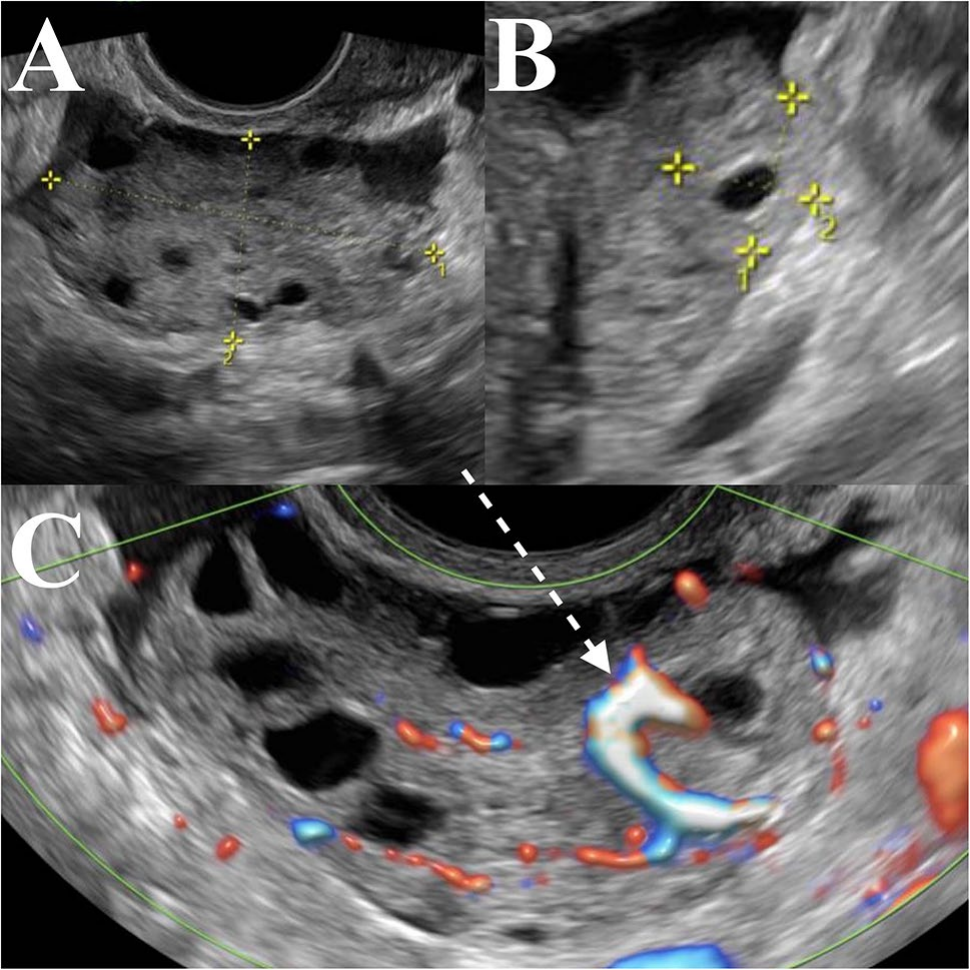
Sonographic follow-up of Case 1 after frozen embryo transfer with hormonal replacement therapy, demonstrating ovarian pregnancy without a corpus luteum. *Dotted arrow indicates the dominant-vessel Doppler sign leading to the ovarian pregnancy. *A GA of 5 + 4, Left ovary; **B** GA of 6 + 0, Left ovary with suspected OP; *C*: GA of 6 + 4, Left ovary with suspected OP and doppler pattern

**Fig. 2:**
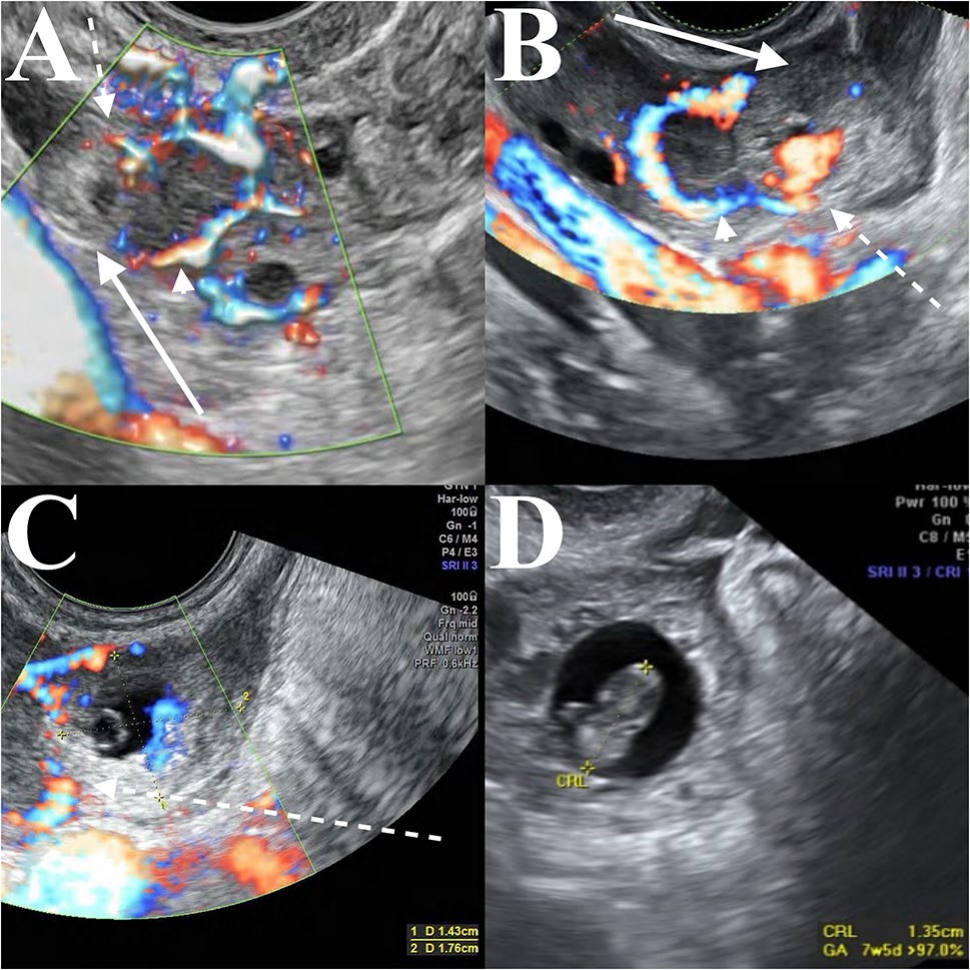
Sonographic images of Cases 3, 5, 7, and 13. *Arrow indicates the ovarian pregnancy. *Arrowheads indicates the corpus luteum. *Dotted arrow indicates the dominant-vessel Doppler sign leading to the ovarian pregnancy. *A Case 3, GA of 4 + 6W, β-hCG 2161, OP with CL; **B** Case 5, GA of 5W, β-hCG 4941. OP with CL; **C** Case 7, GA of 6 + 1W, β-hCG 12787. OP with yolk sac; **D** Case 13, GA of 9 + 4W (CRL = 7 + 5W), β-hCG 19647

Sonographic suspicion was for TEP in 9 cases (60%) and for OP in 8 cases (53.3%). In 2 cases both TEP and OP were considered. Pelvic free fluid was recorded in 12 patients (80%). All six cases that underwent formal ultrasound examination with Doppler demonstrated a distinctive pattern of a single dominant vessel supplying the OP. In the remaining one case with formal ultrasound, Doppler imaging was not recorded. Sonographic findings are shown in Figs. 1, 2.

False-negative cases (OP not suspected preoperatively) occurred in 7/15 cases (46.7%).

**Fig. 3:**
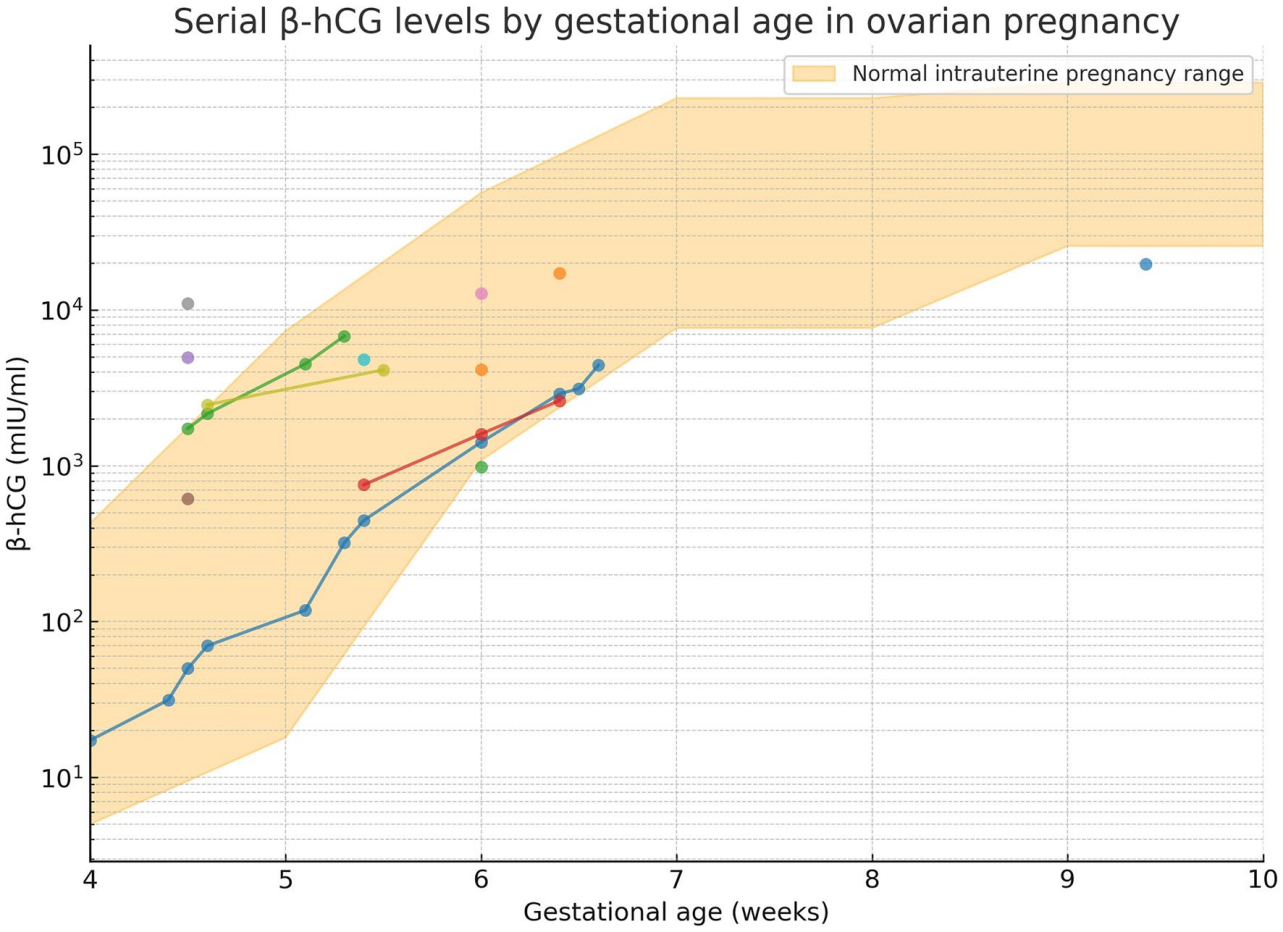
Serial serum β-hCG levels by gestational age compared with the normal intrauterine pregnancy reference range. ^*^Each point represents an individual serum β-hCG measurement. *Points connected by a line correspond to sequential measurements from the same patient, reflecting all values available up to the time of surgery. *The shaded orange area indicates the reference range for β-hCG in normal intrauterine pregnancies [48]. *The y-axis is uses a logarithmic scale

Serial serum β-hCG measurements are illustrated in Fig. 3. While absolute β-hCG values often fell within the expected intrauterine pregnancy range, all cases with more than one measurement showed inappropriate rises, failing to meet the expected doubling pattern.

Higher identification rates were observed in formal ultrasound examinations (6/7, 85.7%) compared with emergency assessments (2/8, 25%); however, this difference should be interpreted cautiously given variability in expertise, equipment, and documentation, particularly in emergency settings. Diagnostic findings are summarized in Table 2.

**Table 2:** Diagnostic Findings and patients outcome

Variable	(*N* = 15)
Gestational age at surgery (weeks)	5.9 ± 1.3
Preoperative β-hCG (mIU/ml)	6436.1 ± 5570.4
Days from first suspicious to surgery	1 ± 2.4
Vaginal bleeding	4 (26.7%)
Abdominal pain	13 (86.7%)
Preoperative hemoglobin (g/dL)	11.9 ± 1.4
Endometrium thickness ≥ 10 mm, *N* = 13	9 (69.2%)
Trilaminar endometrium	0
Corpus luteum recorded in addition	5 (33.3%)
Sonographic suspicion of TEP^a^	9 (60%)
Sonographic suspicion of OP^a^	8 (53.3%)
Yolk sac visible	9 (60%)
Pelvic fluid	
None	3 (20%)
Small\Moderate	8 (53.3%)
Large	4 (26.7%)
EP side on US (Right), *N* = 14^b^	11 (78.6%)
Imaging quality	
Formal US	7 (85.7% diagnosed correctly)
ER OBGYN assessment	8 (25% diagnosed correctly)
Single dominant vessel supplying the OP (N = 6, Formal US with Doppler)	6 (100%)
Estimated blood loss during surgery (ml)	472.1 ± 510.2 (range 10–2000)
Rupture	11 (73.3%)
Hemoperitoneum	11 (73.3%)
Fallopian tube or uterus findings	0
Adhesions	4 (26.7%)
Peritoneal endometriosis	1 (6.7%)
^b^OP side as diagnosed? (N = 14)	14 (100%)
Postoperative day 1 β-hCG decline (%)	59.2 ± 11.7
Ovarian preservation	15 (100%)

*OP* ovarian pregnancy, *TEP* tubal ectopic pregnancy, *US* ultrasound, *ER* emergency room, *OBGYN* obstetrics and gynecology, *β-hCG* beta–human chorionic gonadotropin, *EP* ectopic pregnancy

Results are presented as mean ± SD or n (%) when appropriate

Denominators vary due to missing or non-evaluable data. Percentages are calculated based on available cases for each variable

Formal US = Formal ultrasound examination performed in a dedicated Obstetrics and Gynecology unit with stored images

ER OBGYN assessment = Bedside ultrasound performed by an emergency room Obstetrics and Gynecology physician, documented by report only

^a^Two cases were initially considered in the differential diagnosis for both OP and TEP, and therefore appear in both categories

^b^Refers only to the side of diagnosis (including cases initially misdiagnosed as TEP). One case had no documented sonographic diagnosis side due to the presence of hemoperitoneum and the urgency of intervention

At surgery, the affected ovary was right-sided in 12 of 15 cases (80%). OP rupture occurred in 11 patients (73.3%), and adhesions were identified in 4 (26.7%). Hemoperitoneum was confirmed in 11 cases (73.3%), consistent with preoperative imaging in 10. One patient also had peritoneal endometriosis.

The mean estimated blood loss was 472.1 ± 510.2 ml (range 10–2000 ml, median 300 mL). Postoperative β-hCG decline on day 1 averaged 59.2 ± 11.7%. In all cases, ovarian preservation was achieved. In 14/14 cases intraoperative localization of the affected ovary was correct. One case had no documented sonographic diagnosis side due to the presence of hemoperitoneum and the urgency of intervention.

Surgical findings and outcomes are detailed in Table 2.

A case-level summary of clinical, laboratory, and imaging findings is presented in Supplementary Table S2.

### Narrative literature overview

#### Etiology and prevalence

In 1951, Hertig [8] estimated that OP occurred in approximately 1 in 110 ectopic gestations (~ 1%). Larger studies in the 1980s [9] suggested a prevalence of 3.2% of all EPs. More recent research reports a stable rate of 1–3% [1–3, 10–12].

Although OP prevalence among EP appears stable [12], the overall incidence of EP has steadily increased over the last three decades [3, 11, 13], largely due to greater use of early ultrasound and changes in reproductive health practices. Some OP cases may be underdiagnosed and misclassified as TEP, tubal abortions, or “complex adnexal masses” suitable for expectant or methotrexate (MTX) management without laparoscopic confirmation [12].

Contributors to the rising EP incidence include ART use, delayed childbearing, increased rates of sexually transmitted infections, IUD utilization, and higher frequencies of pelvic surgery. The global increase in cesarean section rates has also led to a rise in cesarean scar pregnancies [11]. Improved ultrasonographic sensitivity further contributes to higher detection rates.

The reported incidence of OP per pregnancy varies widely: ranging from 1 in 1,000 to 1 in 60,000, reflecting both underdiagnosis and regional variation [4, 14–16].

Two main mechanisms are proposed [17, 18]:

- Primary (intrafollicular) OP – fertilization occurs within the follicle, with the gestational sac developing in the ovary at the site of the CL.
- Secondary (extrafollicular) OP – a fertilized ovum from the fallopian tube reimplants on the ovarian surface.

#### Risk factors

A 2022 retrospective case–control study of 146 OP cases matched to TEP and intrauterine pregnancies (IUP) identified ART (OR 2.59, 95% CI 1.25–5.37) and IUD use (OR 2.77, 95% CI 1.74–5.71) as significant risk factors [4]. Similarly, a 2020 series of 79 OP cases among 6,943 EPs found that IUD use and ART increased OP risk, while multiparity was protective [3].

Following in vitro fertilization and embryo transfer, OP accounts for up to 6% of EPs, compared with ~ 3% after spontaneous conception [19, 20]. Recent studies have not confirmed earlier reports linking endometriosis to OP risk [3, 21, 22].

#### Proposed mechanisms

- ART – retrograde embryo migration due to high-volume transfer medium or uterine contractions during difficult transfers, ovarian trauma from oocyte retrieval, and increased sperm access to the ovary after intrauterine insemination.
- IUD – altered tubal motility may promote ovarian implantation [15].

### Clinical presentation

A 2024 retrospective study comparing OP with TEP found that OP was more likely to present with abdominal pain without vaginal bleeding (60% vs 13%; OR 10.0). Additionally, OP was more likely to contain an embryo with cardiac activity (15% vs 2%; OR 8.7), and to present with severe hemoperitoneum (45% vs 8%; OR 9.4). Median intraoperative blood loss was substantially greater (700 mL vs 100 mL). All OPs were managed laparoscopically with ovarian preservation in 95% of cases [15].

Earlier studies reported similar findings, with OP associated with higher β-hCG levels, early rupture, and greater hemodynamic instability than TEP [19, 21]. Due to the ovary’s limited capacity to accommodate a gestation, rupture is common (52–80% of cases) [3, 15, 19, 21].

### Diagnosis

While ultrasonography for EP diagnosis has been available for decades [23], early approaches relied on excluding IUP rather than visualizing extrauterine gestation, leading to frequent “negative laparoscopies” [24].

Ultrasound criteria for OP exist but are under-validated, while some studies report lower preoperative detection rate [3, 7], a recent case–control study demonstrated that 75% of OPs were correctly diagnosed preoperatively, with the remainder misclassified as TEP [15]. Typical features include:

- Location completely or partially within ovarian parenchyma, inseparable from the ovary.
- Gestational sac distinct from any ipsilateral CL.
- Hyperechogenic sac wall, sometimes with mild cystic change.
- Vascularity on Doppler less intense than CL “ring of fire.”

Previous studies have suggested similar diagnostic protocols [25].

Differentiation from a TEP adherent to the ovary [15], a ruptured TEP or CL[3, 26], or a hemorrhagic CL can be challenging. The presence of two CLs [27] or a CL in hormonally supported frozen embryo transfer (FET) cycles (reported in 1.9–7.4% of cases) [28] may further complicate interpretation.

The endometrium in TEP pregnancies often demonstrates a triple-layered pattern [6, 29–31], with a median endometrial thickness of 6.0–13.1 mm [15, 32]; however, literature describing endometrium characteristics in OP is scarce.

MRI can aid diagnosis when ultrasound is inconclusive, showing a gestational sac-like structure within the ovary and normal fallopian tubes [14, 17, 33, 34].

Definitive diagnosis is surgical, based on the Spiegelberg pathological criteria (1878) [35]: intact ipsilateral tube, gestational sac in ovarian position, ovarian ligament connection, and histologic confirmation of ovarian tissue in the sac wall.

### Management

Most OPs require surgery due to rupture risk and heavy bleeding. Conservative laparoscopic excision with maximal ovarian preservation is preferred when feasible [15]. Laparoscopic management was first reported in 1988 [36].

Techniques include wedge resection or cystectomy using monopolar/bipolar electrosurgery [37], ultrasonic device or adjuncts such as vasopressin injection (with 20 IU diluted in 80 mL 0.9% sodium hypochlorite) for hydrodissection and hemostasis [38].

Medical management with systemic or locally injected MTX has been reported [39–42], but its role remains limited due to rupture risk and high vascularity. Criteria have been suggested for the use of systemic MTX if all of the following are met: (1) no signs of hemodynamic compromise; (2) no evidence of blood in the pelvis; (3) pregnancy size < 3.5 cm with no fetal heart activity; and (4) β-hCG < 3,500 IU/L [43]. ASRM guidelines do not recommend MTX as first-line therapy [44].

In a series of 112 OPs, 85 underwent primary surgery, 3 required surgery after failed MTX, and 24 after failed expectant management [21]. Rare reports describe alternative agents such as local injection of etoposide [45].

### Outcomes

In the same series [21], reproductive outcomes among 49 patients with desire of fertility were followed for 3 years:

- 48.9% spontaneous IUP,
- 10.2% IUP via ART,
- 4% recurrent EP,
- 36.7% no conception.

Outcomes did not differ significantly in reproductive outcomes between laparotomy and laparoscopy.

Compared with TEP, OP is associated with greater intraoperative blood loss, higher transfusion rates, and more frequent rupture and hemoperitoneum, but mortality remains rare in settings with rapid surgical access [19].

## Discussion

Ovarian pregnancy remains a diagnostic challenge due to its nonspecific clinical manifestations and similarity to TEP. In this case series, we report 15 histologically confirmed OPs, highlighting their clinical, laboratory, and sonographic features with a narrative literature overview to support earlier recognition and timely management.

In our cohort, two-thirds of patients (10/15) had identifiable risk factors, including IUD use (n = 7), ART (n = 2), and endometriosis (n = 1). These findings parallel recent case–control studies that identified IUD use (OR ~ 2.8) and ART (OR ~ 2.6) as independent risk factors [3, 4]. Endometriosis has been inconsistently reported in the literature, with more recent studies not confirming a significant association [3, 21, 22].

Abdominal pain was the most frequent presenting symptom in our series (86.7%), while vaginal bleeding occurred in only 26.7%. These findings echo those of a 2024 case–control study, which showed OP to be significantly more likely than TEP to present with abdominal pain without bleeding (60% vs 13%) [15].

Absolute β-hCG values in our patients often overlapped with intrauterine pregnancy ranges, which was not consistent with earlier series that reported higher β-hCG levels [19, 21]. However, in all cases with serial measurements, the rise was inappropriate, failing to achieve the expected doubling. In addition, we observed a consistent and sharp postoperative β-hCG decline, reflecting resolution of trophoblastic tissue.

### Sonographic findings

Our cohort confirms the central role of ultrasound, while also highlighting its limitations. The hyperechoic circular ring at the lateral aspect of the ovary was a consistent feature, with yolk sac visualization in 60% of cases. However, in 4 of the 6 cases with a visible yolk sac, the sonographic impression was initially of TEP, underscoring the diagnostic overlap between OP and TEP.

**Fig. 4:**
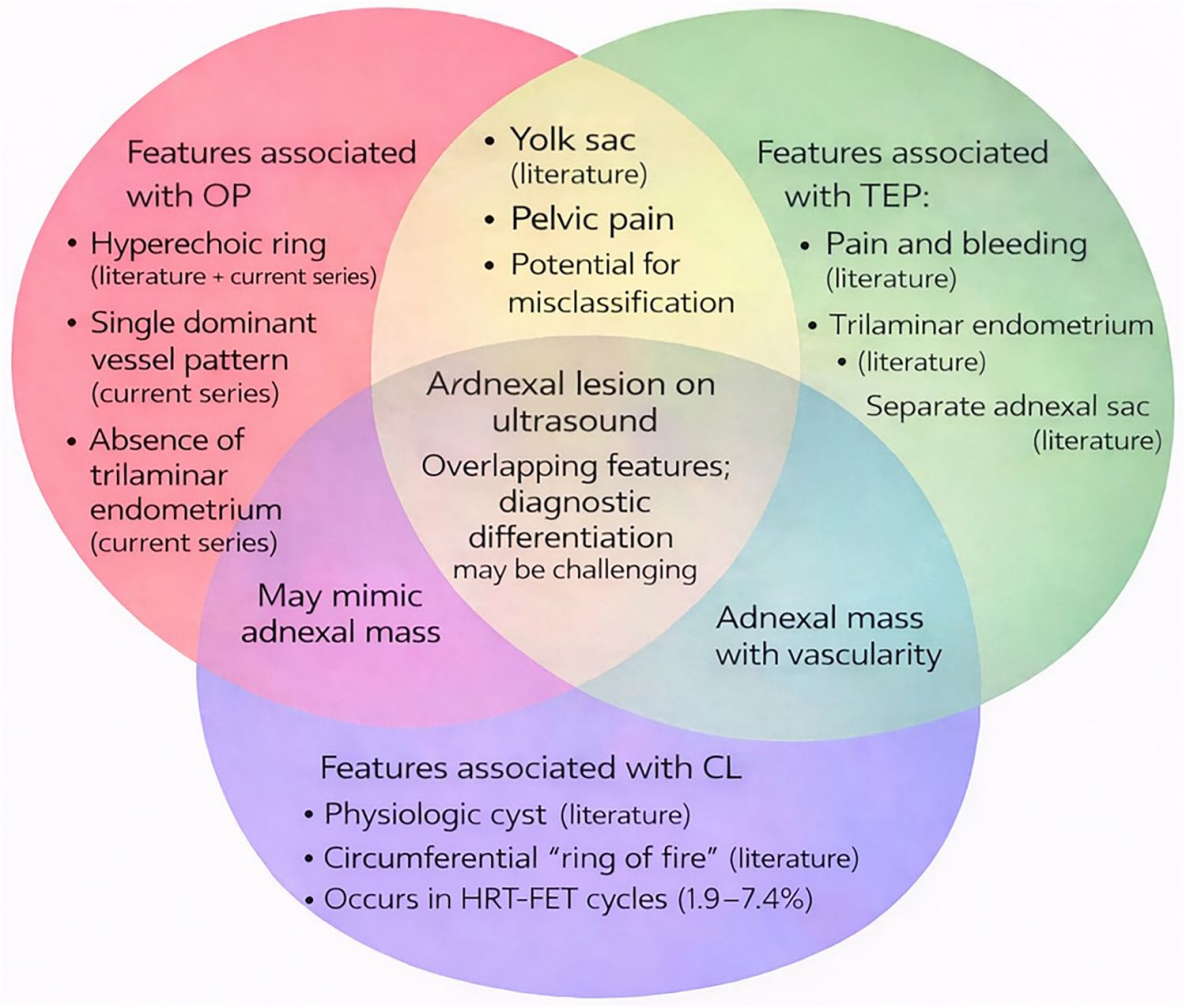
Conceptual diagram illustrating the diagnostic overlap and distinguishing features of ovarian pregnancy, tubal ectopic pregnancy, and corpus luteum. Conceptual illustration of overlapping and distinguishing sonographic and clinical features of ovarian pregnancy (OP), tubal ectopic pregnancy (TEP), and corpus luteum (CL). Features are derived from the current case series and the literature and are presented for illustrative purposes only, not as validated diagnostic criteria. Labels indicate whether features were observed in the current series or reported in the literature. *US, ultrasound; HRT-FET, hormone replacement therapy–frozen embryo transfer

A comparative summary of the diagnostic and clinical characteristics of OP, TEP, and CL is provided in Table 3, while Fig. 4 illustrates their overlapping and distinguishing sonographic features.

**Table 3:** Comparative Diagnostic and Clinical Features of Ovarian Pregnancy (OP), Tubal Ectopic Pregnancy (TEP), and Corpus Luteum (CL)

Feature	Ovarian Pregnancy (OP)	Tubal Ectopic Pregnancy (TEP)	Corpus Luteum (CL)
Prevalence	1–3% of ectopic pregnancies (L)	~ 95–97% of ectopic pregnancies (L)	CLs can evolve simultaneously (L). A CL occurs in 1.9–7.4% of HRT-FET cycles (L)
Risk factors	IUD use 46.7% (C); OR 2.77 (L). ART 13.3% (C); OR 2.59 (L). Endometriosis: 1 case (C), not confirmed (L)	PID, prior tubal surgery, ART, IUD (L)	
Clinical presentation	Abdominal pain 86.7% (C); vaginal bleeding 26.7% (C). OP more often pain without bleeding: 60% vs 13% in TEP (L)	Abdominal pain and vaginal bleeding > 70% (L)	
β-hCG pattern	Overlaps IUP (L, C); rise inappropriate (C); sharp postoperative decline (C)	Abnormal rise, lower than IUP (L)	
Endometrium	Thick > 10 mm in 69.2% (C), no trilaminar (C). Literature: scarce (L)	Often trilaminar; median 6–14 mm[47] (L)	
Ultrasound	Hyperechoic ring in all cases (C); yolk sac in 60% (C); CL also noted in some (C). Sac inseparable from ovary, distinct from CL (L); Thick endometrium (9/13), but none exhibited a trilaminar pattern (13/13) (C)—Warrant further study	Adnexal sac/mass separate from ovary (L)	Ovarian cyst, hypoechoic/complex, central clot possible (L)
Doppler	Single dominant vessel (warrant further study) (C); less vascular than CL “ring of fire” (L)	Peritrophoblastic “ring of fire” (L)	Circumferential “ring of fire” (L)
Misclassification	Often mistaken for TEP/CL (L)		May mimic OP or TEP (L)
Rupture & Hemoperitoneum	Rupture 73.3% (C); median blood loss 300 mL (C). Literature: rupture 52–80%, blood loss ~ 700 mL vs 100 mL in TEP (L)	Rupture and bleeding common (L)	
Management	All surgical with ovarian preservation (C). Laparoscopic wedge/cystectomy preferred (L). MTX limited, not first-line (L)	Surgery: salpingectomy/salpingostomy; MTX if criteria met (L)	

*OP* ovarian pregnancy, *TEP* tubal ectopic pregnancy, *CL* corpus luteum, *IUD* intrauterine device, *ART* assisted reproductive technology, *PID* pelvic inflammatory disease, *IUP* intrauterine pregnancy, *MTX* methotrexate, *HRT-FET* hormone-replacement therapy frozen embryo transfer, *β-hCG* beta–human chorionic gonadotropin, *C* current series (*N* = 15), *L* literature

This aligns with prior studies that reported preoperative diagnostic accuracy rates of ~ 75%, with the remainder misclassified as TEP [15]. In our series, suspicion of OP was raised in 53.3%, slightly lower than published rates, but diagnostic accuracy was far higher in formal ultrasound units (85.7%) compared with emergency room evaluations (25%); however, this comparison is confounded by differences in expertise, equipment, and documentation, and should therefore be interpreted cautiously.

Differentiating OP from CL remains a key challenge. In our series, all formal ultrasound examinations with Doppler records (6/6) demonstrated a distinctive pattern of a single dominant vessel supplying only part of the lesion, unlike the circumferential “ring of fire” typically seen in a CL. This Doppler finding represent a hypothesis-generating feature requiring validation in larger studies. An analogous concept has been reported in TEP, where Ramanan and colleagues described the *leash sign*-a vascular pedicle supplying the ectopic mass-with excellent diagnostic accuracy (sensitivity 100%, specificity 99%, positive predictive value 95%, negative predictive value 100%) [46]. Diagnostic complexity is further heightened by the rare occurrence of two CLs [27] or CLs in hormonally supported FET cycles (1.9–7.4% of cases) [28].

Interestingly, none of our cases with endometrial documentation (13/13) demonstrated a trilaminar endometrial pattern, in contrast to reports of such findings in TEP [6, 29–31]. Literature describing endometrium in OP is limited, but our data suggest this feature may be unreliable in differentiating OP. In a considerable number of cases, the presence of an IUD prevented clear visualization of the endometrial pattern; in other cases, fluid accumulation within the uterine cavity was observed.

OP rupture was observed in 73.3% of our cases, consistent with the high rupture rates (52–80%) reported previously [3, 15, 19, 21], reinforcing the high bleeding risk. The median estimated blood loss was 300 mL (range 10–2000 mL), lower than the median 700 mL reported in a recent case–control study [15].

As with most published series [15, 21], all our cases underwent surgical management (due to the requirement for histological confirmation), and ovarian preservation was achieved in all.

### Strengths and limitations

Strengths include the multicenter design, histologic confirmation, and integration of a narrative literature overview. In addition, the availability of high-quality sonographic images provides visual reinforcement of the diagnostic features described.

Limitations include the retrospective design, relatively small sample size, and reliance on available imaging and operative reports. Diagnostic interpretation was influenced by operator expertise and the quality of documentation, particularly in emergency settings. The interpretation of sonographic features is limited by incomplete imaging availability and variability in documentation inherent to the retrospective design. While our findings add to the growing body of literature, larger prospective multicenter studies are needed to validate the observed sonographic features, quantify diagnostic accuracy, and further delineate the role of β-hCG dynamics in OP diagnosis.

## Conclusion

This case series highlights clinical and sonographic characteristics of OP. We observed a Doppler pattern suggestive of a single dominant vessel and absence of a trilaminar endometrium, which may represent hypothesis-generating findings requiring validation in larger studies. The inclusion of representative sonographic images and a narrative literature overview provide clinicians and researchers with tools and context for improved diagnosis and support future investigation.

## Supplementary Information

Below is the link to the electronic supplementary material.

Supplementary file1 (DOCX 27 KB)

Supplementary file2 (XLSX 15 KB)

## Data Availability

The data from this study is available from the corresponding author upon a reasonable request and following approval of the institutional review board.
