# Supplementary material for: Ovarian pregnancy: clinical characteristics, diagnostic challenges, and sonographic features—a multicenter case series with narrative literature overview
Source: Arch Gynecol Obstet. 2026 May 13;313(1):217. doi: 10.1007/s00404-026-08460-w (PMC13337687; doi:10.1007/s00404-026-08460-w)
Supplement: Supplementary file 1 — Supplementary file1 (DOCX 27 KB) [file 404_2026_8460_MOESM1_ESM.docx]

# Supplementary Table 1. Availability and Reasons for Missing or Non-Evaluable Data

| Variable | Available (n/N) | Missing (n) | Reason for missing/non-evaluable |
| --- | --- | --- | --- |
| Formal ultrasound imaging | 7/15 | 8 | Emergency assessments without stored images |
| Doppler evaluation | 6/15 | 9 | 8 missing due to emergency assessments without stored images; 1 formal ultrasound without Doppler performed |
| Endometrial pattern and thickness | 13/15 | 2 | 7 cases with formal ultrasound and stored images; 6 cases based on text descriptions without images |
| Sonographic diagnosis (OP vs TEP) | 15/15 | 0 | 7 cases with formal ultrasound and stored images; 8 cases based on text descriptions without images |
| Side of ectopic pregnancy | 14/15 | 1 | Hemoperitoneum, urgent surgery |

**Abbreviations:** OP, ovarian pregnancy; TEP, tubal ectopic pregnancy;
